# Blockade of Renin Angiotensin System Increased Resistance to STZ-Induced Diabetes in Rats with Long-Term High-Fat Diet

**DOI:** 10.1155/2012/618923

**Published:** 2012-11-12

**Authors:** Xin Li, Li Yuan, Jin Li, Hailing Li, Suosuo Cheng

**Affiliations:** Department of Endocrinology, Zhongnan Hospital, Wuhan University, 169 Donghu Road, Wuhan 430071, China

## Abstract

This study aimed to investigate whether rennin-angiotensin system (RAS) blockade through telmisartan would increase the resistance to streptozotocin- (STZ-) induced diabetes in insulin resistance rats. There were sixty Wistar rats that were divided into four groups: normal control (NC), high-fat diet (HF), high-fat diet plus STZ injection (HF+S), and high-fat diet plus STZ injection and telmisartan intervention (HF+S+T). Five rats were chosen randomly and respectively from groups NC and HF to undergo a hyperinsulinemic euglycemic clamp. Another five rats were selected randomly from the four groups, respectively, for intravenous injection insulin releasing test (IVIRT), and the other five rats for pancreas specimens used in islet cell immunohistochemistry staining (stained for insulin, NF-**κ**B, and caspase-3), islet cell apoptosis staining, and reverse transcription PCR (AT1R and IL-1 beta). There was a significant difference of overt diabetes incidence between groups HF+S+T and HF+S (*P* < 0.05). Furthermore, inflammatory markers and islet cell apoptosis were found to be significantly reduced in group HF+S+T compared with group HF+S (all *P* < 0.01 or *P* < 0.05). Overall, telmisartan-treated rats were found to have reduced RAS activity, increased resistance to STZ-induced diabetes, reduced inflammatory markers, and improvement of islet cell function and morphology.

## 1. Introduction

Pancreatic beta-cell dysfunction and insulin resistance are the hallmarks of type 2 diabetes. Normal beta cells can compensate for insulin resistance by increasing insulin secretion and/or beta-cell mass, but insufficient compensation leads to the onset of glucose intolerance. Once hyperglycemia becomes apparent, beta-cell function gradually deteriorates and insulin resistance aggravates [1]. So, to explore the strategy to protect islet function and prevent or delay the onset of overt diabetes in prediabetes stage is of great importance. 

Recently, an intrinsic renin angiotensin system (RAS) has been found in the endocrine pancreas [2, 3]. Several large clinical trials [4] have reported that blockade of RAS may prevent or delay the development of overt diabetes in “at-risk” patients. The NAVIGATOR study (Nateglinide and Valsartan in Impaired Glucose Tolerance Outcomes Research) showed that to use of valsartan for 5 years, along with lifestyle modification, led to a relative reduction of 14% in the incidence of diabetes [5], indicating that RAS blockade may ameliorate the glucose metabolism and islet function of patients with impaired glucose tolerance (IGT). 

Inflammation has been considered as an important pathophysiological mechanism in the development of insulin resistance. Many inflammatory factors, such as interleukin-1 (IL-1), tumor necrosis factor (TNF)-*α*, monocyte chemoattractant protein (MCP)-1, have been found in islets [6]. Angiotensin II has been shown to trigger inflammation through inducing oxidative stress which upregulates inflammatory mediators [7]. So this study aimed to investigate whether RAS blockade through telmisartan would increase the resistance to STZ-induced diabetes in rats of insulin resistance. 

## 2. Research Design and Methods

### 2.1. Animal Feeding and Treatment

Sixty healthy male Wistar rats, 8 weeks of age, were placed in a room with controlled lighting (12 hours light/dark cycle) and regulated temperature (18°C–25°C) and humidity. All rats were fed with regular chow (protein 21%, carbohydrate 55%, fat 6%, and total energy 15.36 kJ/g) for 2 weeks to be adapted for the environment. Fifteen rats were randomly selected as normal control group (NC, *n* = 15), which were fed regular chow throughout the study. The remaining rats were fed with high-fat diet which consisted of regular feedstuff, sucrose, lard, fresh egg, and milk power (protein 16%, carbohydrate 38%, fat 46%, and total energy 20.54 KJ/g). After high-fat diet feeding for 16 weeks, fifteen rats were selected randomly as high-fat diet plus telmisartan intervention (10 mg/kg per day [8]) and STZ intraperitoneal injection (given 8 weeks later) group (HF+T+S, *n* = 15). The remained 30 high-fat diet feeding rats were divided into high-fat diet control group (HF, *n* = 15) and high-fat plus STZ intraperitoneal injection group (HF+S, *n* = 15), which, together with FS group, was given an intraperitoneal injection of streptozotocin (20 mg/kg) in 0.1 mol/L citrate-buffered saline (Table 1). The body weight of all rats was measured weekly during the study and the systolic blood pressure was detected by tail-cuff plethysmography.

### 2.2. Hyperinsulinemic Euglycemic Clamp

Five rats selected randomly from NC group and HF group, respectively, were anesthetized with pentobarbitone (80 mg/kg) after over-night fasting. Both sides of femoral veins were exposed and inserted by a catheter for infusion of glucose and insulin, respectively. Another catheter was inserted into the femoral artery for blood sampling. Rats were kept quiet for 30 minutes, then a 120-minute hyperinsulinemic euglycemic clamp was performed according to reports [9]. In brief, insulin was infused at a constant rate of 1.67 mU/kg per minute and the arterial blood glucose concentration was clamped at the basal fasting level by infusing glucose at variable rates. Under the hyperinsulinemic conditions, the steady glucose infusion rate (GIR) required to maintain euglycemia (usually calculated between 60–120 min) is a standard measure of the whole-body insulin sensitivity. Hyperinsulinemic euglycemic clamp was not done in rats of HF+S, and HF+T+S group for this test was done just to compare the level of insulin resistance after long term high-fat diet feeding.

### 2.3. Intravenous Glucose Tolerance Test and Intravenous Insulin Releasing Test

Another 5 rats selected randomly from the four groups were given intravenous glucose tolerance test (IVGTT) to assess the pancreatic beta-cell function. Animals were fasted overnight before the test, and anesthetized with sodium pentobarbitone (80 mg/kg) through intraperitoneal injection. The left carotid artery and right carotid vein were cannulated. A bolus of glucose load (1.0 g/kg) was injected through the right carotid vein. Rats were kept quiet for 30 minutes, then 1.0 mL blood was sampled at 0 min (before glucose injection) and 0.3 mL blood at 2, 5, 10, 30, 60 min after glucose injection. The cannulas were flushed with saline between sampling. Plasma was collected by centrifuge. Plasma glucose was analyzed by the glucose oxidase method. Plasma insulin was analyzed by radioimmunoassay. Area under the curve of glucose (AUCG) and area under the curve of insulin (AUCI) were calculated by trapezoidal method.

### 2.4. Islet Immunohistochemistry 

Pancreas specimens from the five rats selected randomly from each group were fixed in 4% chilled paraformaldehyde and embedded in paraffin. Sections were mounted on glass slides, deparaffinized, and processed for immunohistochemistry staining. The pancreatic islets were stained for insulin, nuclear factor kappa B (NF-*κ*B), and caspase-3 by certain anti-rat primary antibodies in suitable concentrations. The slides were then incubated overnight at 4°C. After washing with phosphate buffered saline (PBS), the secondary antibody was applied for 30 min at 37°C. And by the same method, the third antibody was applied. DAB/H_2_O_2_ staining was used at last. All sections were analyzed randomly and double-blindly with light microscopy in 400 magnifications, and pictures were captured and evaluated by a computer image analysis system. 

Insulin staining was used to assess the change of islets morphology and insulin concentration in beta cells. The value of islet area, insulin staining area, and gray scale were detected by an image analysis system. The natural logarithm of the reciprocal of gray scale of intra-islet insulin positive staining was calculated as intra-islet insulin relative concentration (IRC), which represented the insulin reserve in endochylema of beta cells. The relative content of NF-KB (NRC) and caspase-3 (CRC) which represented the inflammation and apoptotic signal molecule level intral islets, respectively, was evaluated in the same way. 

### 2.5. Islet Cell Apoptosis

Islet cell apoptosis was detected by transferase-mediated dUTP nick-end labeling (TUNEL) staining. The number of TUNEL-positive cells intra unit islet area under the high power field was calculated to represent the level of islet apoptosis.

### 2.6. Semiquantitative Reverse Transcription Polymerase Chain Reaction (RT-PCR)

The relative expression level of angiotensin II receptor type 1 (AT1R) and interleukin-1*β* (IL-1*β*) mRNA, which represented the activity of RAS and inflammation, was evaluated by RT-PCR. Fresh pancreas isolated from rats was digested by type IV collagenase (0.5 mg/mL), and pancreatic islet cells were extracted. Then total RNA was isolated with Trizol. cDNAwas synthesized with a reverse-transcription reaction and was cloned by PCR. PCR products were cataphoresised in 2% agarose gel. The gray scale of strap was observed under long wavelength UV and taken pictures. The ratio of target gene PCR product gray scale to *β*-actin reflected the relative gene expression level.

### 2.7. Statistical Analysis

All data were shown as means ± SE. Differences between mean values of variables were compared statistically by ANOVA. Differences between incidence of STZ-induced diabetes were compared statistically by chi-square test. All statistics were performed using SPSS 11.0 software. *P* < 0.05 was considered as statistically significant.

## 3. Results

### 3.1. Animal Model

There was no obvious difference in body weight among the three high-fat diet feeding groups, HF, HF+S, and HF+T+S group, which were all higher than that in NC group (all *P* < 0.01). One week after STZ injection, the plasma glucose was measured. Rats with randomized plasma glucose ≥16.7 mmol/L twice not in one day were considered as diabetic rats. The incidence of STZ-induced diabetes was 80% and 33% in HF+S and HF+T+S group, respectively (*P* = 0.025). The mean fasting blood glucose (FBG) of HF+S group was higher than that in HF+T+S group ((17.5 ± 5.1) mmol/L versus (13.2 ± 4.7) mmol/L, *P* < 0.05). When the study finished, the blood pressure of HF group was higher than NC group, but there was no significant difference between every two groups. 

### 3.2. Insulin Sensitivity

The whole-body insulin sensitivity was assessed by glucose infusion rate (GIR). GIR in HF group was (5.32 ± 0.90) mg·kg^−1^·min^−1^, significantly lower than that in NC group ((7.80 ± 0.51) mg·kg^−1^·min^−1^) (*P* < 0.01), indicating the presence of insulin resistance in rats with long-term high-fat diet. 

### 3.3. Islet Function

The islet function was evaluated by intravenous glucose tolerance test (IVGTT) and intravenous insulin releasing test (IVIRT). The fasting blood glucose (FBG) of HF+S group was higher than that in HF+T+S group as shown above. FBG of the two STZ-treated groups were both higher than that in NC or HF group (all *P* < 0.01), while there was no significant difference in FBG between NC and HF group. AUCG from 0 to 60 min (AUCG_0–60_) of HF, HF+S, and HF+T+S group were all higher than that in NC group (all *P* < 0.01). AUCG_0–60_ of HF+S group was higher than that in HF+T+S group (*P* < 0.01), which was higher than that in HF group (*P* < 0.01).

**Figure 1:**
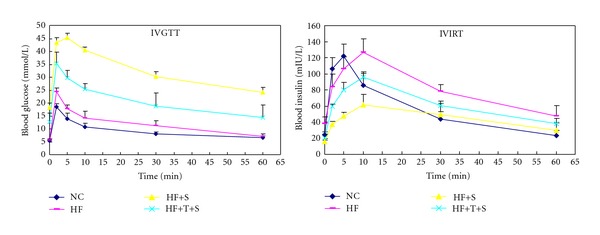
Curve of IVGTT and IVIRT. NC: normal control group; HF: high-fat diet group; HF+S: high-fat diet plus STZ intraperitoneal injection group; HF+T+S: high-fat diet plus telmisartan intervention and STZ intraperitoneal injection group.

The fasting plasma insulin of HF group was (36.18 ± 1.91) mIU/L, which was as 1.19 folds as that in NC group (*P* < 0.01). And the first-stage insulin releasing peak after glucose load was delayed, together with the decreased AUCI_0–10_ and the increased AUCI_10–60_ compared with that in NC group. AUCI_0–10_ and AUCI_0–60_ of HF+T+S group were increased by 61.2% and 49.9%, respectively, (all *P* < 0.01) compared with that in HF+S group (all *P* < 0.01), Figure 1, Table 2.

### 3.4. Islets Morphology and Insulin Content in Beta Cells Endochylema

**Figure 2:**
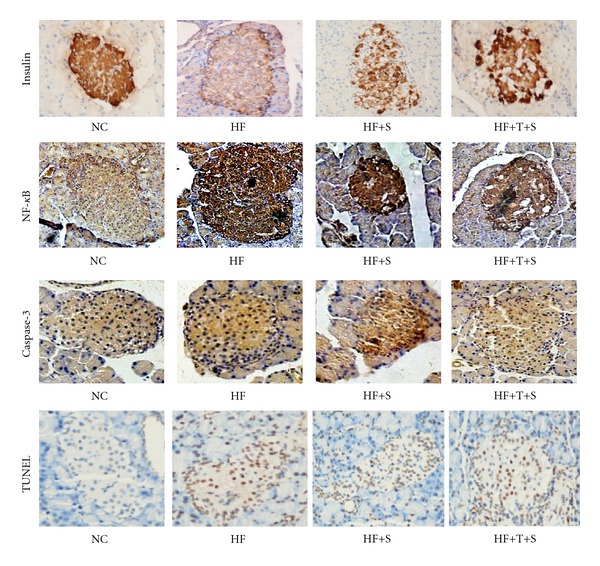
Immunohistochemistry staining of insulin, NF-*κ*B, caspase-3, and TUNEL of pancreas. After fixing and embedding, every pancreas was sliced 5 sections for each index and was stained with certain antibodies. Then the sections were numbered at random and selected 6–8 fields to view and analyze quantitatively. The figures were insulin, NF-*κ*B, caspase-3, and TUNEL, respectively, 10 × 40 amplified, the tan domain was positive staining. NC: normal control group; HF: high-fat diet group; HF+S: high-fat diet plus STZ intraperitoneal injection group; HF+T+S: high-fat diet plus telmisartan intervention and STZ intraperitoneal injection group.

The insulin immunohistochemistry staining was used to evaluate the islets morphology and insulin content in beta cells endochylema. In HF group, most islets were significantly enlarged, while insulin relative content (IRC) (−5.20 ± 0.09) was decreased obviously compared with that in NC group (−4.14 ± 0.14) (*P* < 0.01), indicating the reduced insulin reserve in HF group. Insulin-positive cell density (ICD) in HF group was lower than that in NC group, but the difference was not significant. IRC and ICD in HF+T+S group was higher than that in HF+S group (all *P* < 0.01 or *P* < 0.05), Figure 2, Table 3.

### 3.5. Inflammation Intra Islet

**Figure 3:**
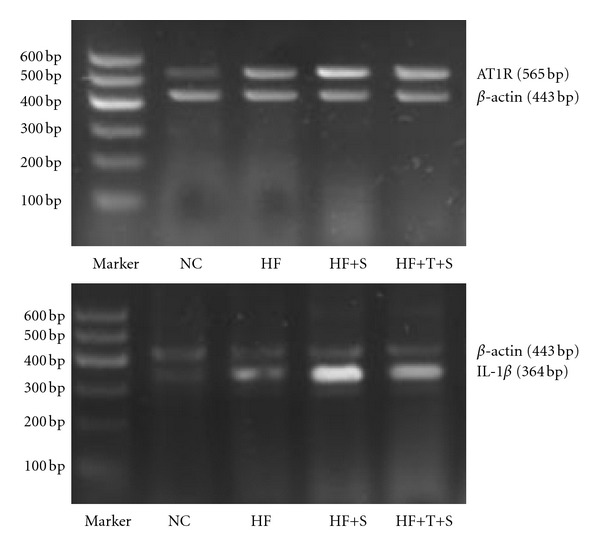
Comparison of AT1R and IL-1*β* mRNA by semiquantitative RT-PCR. NC: normal control group; HF: high-fat diet group; HF+S: high-fat diet plus STZ intraperitoneal injection group; HF+T+S: high-fat diet plus telmisartan intervention and STZ intraperitoneal injection group.

The expression of inflammatory factor, IL-1*β*, was detected by RT-PCR. The relative expression value of IL-1*β* in HF group was 1.75 times higher than that in NC group, indicating obvious inflammation occurred in islets of insulin resistance rats. While the expression level of IL-1*β* mRNA in STZ-treated animals was all increased compared with that in HF group, its expression was relatively lower in HF+T+S group (all *P* < 0.01). The relative content of NF-*κ*B (NRC), a crucial transcription factor of inflammation, apoptosis, proliferation, and so forth, was detected by immunohistochemistry technique. The variance tendency of NRC among the four groups was at equal pace with that of IL-1*β*, indicating the coactivation or exactivation effect of the two factors, Figures 2 and 3, Table 3.

### 3.6. Apoptotic Molecule and Cell Apoptosis in Islets

 Caspase-3, which has tight relationship with NF-*κ*B, is the key molecule in apoptotic signal transduction. The relative content of caspase-3 (CRC) was detected by immunohistochemistry technique to represent the activity of apoptotic signal. In comparison with NC group, CRC in islets of HF group was increased by 19.1% (*P* < 0.01). CRC in HF+T+S group was decreased significantly compared with that in HF+S group, which was higher than that in HF group (all *P* < 0.01 or *P* < 0.05) (Figure 2, Table 2).

 TUNEL was used to identify apoptotic cells within islet boundaries. The number of apoptotic cells intra islet in unit area was significantly greater in HF group (0.72 ± 0.10) than that in NC group (0.21 ± 0.05) (*P* < 0.01). Apoptotic cells in unit islet area of HF+T+S group [(0.84 ± 0.06)/um^2^] was decreased significantly in compared with that in HF+S group ((0.98 ± 0.04)/um^2^), which was higher than that in HF group (all *P* < 0.01 or *P* < 0.05), Figure 2, Table 2.

### 3.7. Expression of RAS

The relative expression of AT1R, which has critical role in mediating the effects of RAS, was detected by RT-PCR. In comparison with NC group, the relative content of AT1R in islet of HF group was increased by 1.21 times (*P* < 0.01). AT1R expression in HF+T+S group was decreased significantly by 11.8% in compared with that in HF+S group, which was higher than that in HF group by 21.1% (all *P* < 0.01 or *P* < 0.05), Figure 3, Table 2.

## 4. Discussion

Activation of RAS has a pivotal role in the pathogenesis of diabetic complications. However, recent evidence indicated that it may also contribute to the development of diabetes. A number of experimental and clinical investigations have supported the notion that angiotensin II contributes to the progression of insulin resistance. Infusion of angiotensin II has been shown to induce the reduction of insulin sensitivity in vivo, while administration of AT1 receptor blockers (ARB) or ACE inhibitors (ACEI) significantly improved insulin sensitivity in hypertensive patients [10]. Several large clinical trials [11] have demonstrated that blockade of RAS protected against the development of diabetes in “at-risk” patients. Multiple researches have shown that blockade of RAS may protect islets function in diabetic animals via ameliorating oxidative stress, fibrosis and apoptosis [12]. Islet function during prediabetes stage has been shown to be abnormal, such as the delayed “first-stage” insulin releasing after glucose load, impaired glucose tolerance, and so forth [13]. So this study was designed to investigate whether RAS blockade may prevent or delay the onset of overt diabetes in insulin resistance rats through improving islet function. 

In this research, insulin resistance was induced by long-term high-fat diet, which has been used extensively and effectively, and has been confirmed valid again in our study by hyperinsulinemic euglycemic clamp. Meanwhile, the first-stage insulin release peak was delayed with decreased AUCI from 0 to 10 min and increased AUCI from 10 to 60 min during IVIRT, which is the typical feature of the islet function in insulin resistance state. We also found that insulin content intra beta cells was decreased obviously, indicating the reduced insulin reserve, which may be the reason for the delayed first-stage insulin releasing peak. To investigate the effect of RAS blockade on the incidence of overt diabetes, low-dose STZ was given to the rats after long-term high-fat diet fed with or without telmisartan. Considering that our rats had been fed high-fat diet for 24 weeks, there may be severe insulin resistance and islet dysfunction compared with those just fed 8 or 12 weeks, so the dose of STZ was selected as 20 mg/kg, lower than that used after 8 or 12 weeks high-fat diet feeding generally, that was 30 mg/kg or 35 mg/kg. The model creation achievement ratio in rats with high-fat diet was 80%, similar to other reports. But the incidence of overt diabetes in rats with telmisartan intervention was only 33%, indicating the increased resistance to STZ-induced diabetes in rats with long-term high-fat diet through the blockade of RAS. After the treatment of STZ, the insulin release peak and AUCI from 10 to 60 min during IVIRT were all decreased, indicating that STZ damaged islet beta cell function severely. We also found that the AUCI was higher in HF+T+S group than that in HF+S group, while FBG and AUCG was lower than that in HF+S group, indicated that RAS blockade may ameliorate glucose as a result of the improved islet function.

Inflammation has been considered as an important aetiological factor in the development of insulin resistance [14]. In this study, the increased content of IL-1*β* was also found in islets of insulin resistance rats, which was increased more after STZ treatment, indicating an obvious inflammation damage intra islets. Many reports have shown that RAS has tight relationship with inflammation, and blockade of RAS may protect organ function via its anti-inflammatory effect, such as in nonalcoholic steatohepatitis (NASH) [15] and endothelial cells by suppressing ROS generation [16]. In our study, the expression of IL-1*β* in rats with telmisartan plus STZ treatment was significantly lower than that in rats with only STZ treatment, suggesting the blockade of RAS may increase the resistance to STZ-induced diabetes partially through the alleviation of inflammatory damage.

 Apoptosis was the result of all the damage factors mentioned above and the main reason for the loss of islet function. In this study, we found that the overactivated apoptotic signal and increased apoptotic cells may be induced by high concentration of FFAs and inflammation. STZ increased the apoptosis of islet beta cells as shown in our results and other papers. The level of apoptosis in islets was lower in the rats with telmisartan intervention than those just given STZ, indicating that RAS blockade may reduced apoptosis of beta cells, and this may explain partly why the the rats had better islet function and lower overt diabetes incidence after telmisartan treatment.

In whole, our research found that with the development of overt diabetes, the abnormalities of islets morphology and function deteriorated, concomitant with the increased activity of RAS, inflammation, and apoptosis, which may interact tightly with each other. With the intervention with telmisartan in early phase, the adverse factors mentioned above were all ameliorated with the improvement of islets morphology and function. So we concluded that blockade of RAS may have protective effects on islet morphology and function in pre-diabetes phase and increase the resistance to STZ-induced diabetes via the amelioration of inflammation and apoptosis. 

## Figures and Tables

**Table 1:** Animal feeding and treatment.

2 weeks	Regular chow (*n* = 60)			
16 weeks	High fat diet (*n* = 45)		
8 weeks	Plus telmesartan (*n* = 15)	High fat diet (*n* = 30)		Regular chow (*n* = 15)
2 weeks	Plus STZ (*n* = 15)	Plus STZ (*n* = 15)	High fat diet (*n* = 15)

STZ: streptozotocin.

**Table 2:** Results of hyperinsulinemic euglycemic clamp, IVGTT, and IVIRT ($\bar{x}\pm s$).

Group	*n*	GIR (mg·kg^−1^·min^−1^)	AUCG_0–60_ (mmol·L^−1^·min^−1^)	AUCI_0–10_ (mIU·L^−1^·min^−1^)	AUCI_10–60_ (mIU·L^−1^·min^−1^)	AUCI_0–60_ (mIU·L^−1^·min^−1^)
NC	5	7.80 ± 0.51	57.12 ± 5.38	277.29 ± 5.46	94.57 ± 2.35	371.85 ± 5.97
HF	5	5.32 ± 0.90*	73.63 ± 6.69*	264.46 ± 3.51*	159.67 ± 7.98*	424.13 ± 9.20*
HF+S	5	ND	180.79 ± 7.31^∗#^	122.84 ± 11.84^∗#^	94.65 ± 24.67^#^	217.49 ± 35.67^∗#^
HF+T+S	5	ND	122.68 ±18.44^∗#∇^	198.13 ± 13.12^∗#∇^	124.88 ± 7.80^∗#★^	326.01 ± 19.68^∗#∇^

NC: normal control group; HF: high-fat diet group; HF+S: high-fat diet plus STZ intraperitoneal injection group; HF+T+S: high-fat diet plus telmisartan intervention and STZ intraperitoneal injection group.

GIR: glucose infusion rate; AUCG: area under the curve of glucose; AUCI: area under the curve of insulin.

^●^ *P* < 0.05; *P < 0.01 as compared with NC group; ^▲^ *P* < 0.05, ^#^<0.01 as compared with HF group; ^★^<0.05, ^∇^<0.01 as compared with HF+S group.

**Table 3:** Quantitive analysis of RT-PCR and immunohistochemistry results ($\bar{x}\pm s$).

Group	*n*	ARC	ILRC	IRC	ICD	NRC	CRC	AC
NC	5	0.73 ± 0.20	0.57 ± 0.13	−4.14 ± 0.14	2.51 ± 0.26	−5.02 ± 0.19	−5.18 ± 0.11	0.21 ± 0.05
HF	5	1.61 ± 0.19*	1.57 ± 0.22*	−5.20 ± 0.09*	2.37 ± 0.08	−3.99 ± 0.33*	−4.19 ± 0.12*	0.72 ± 0.10*
HF+S	5	1.95 ± 0.11^∗#^	1.93 ± 0.17^∗#^	−5.42 ± 0.07^∗#^	1.45 ± 0.14^∗#^	−3.40 ± 0.14^∗#^	−3.84 ± 0.11^∗#^	0.98 ± 0.04^∗#^
HF+T+S	5	1.72 ± 0.14^∗★^	1.67 ± 0.11^∗★^	−5.33±0.05^∗▲★^	1.78 ± 0.12^∗#∇^	−3.62 ± 0.12^∗▲★^	4.02 ± 0.07^∗▲★^	0.84 ± 0.06^∗▲∇^

ARC: AT1R mRNA relative content; ILRC: IL-1*β* mRNA relative content; IRC: insulin raltive concentration; ICD: insulin positive cell density; NRC: NF-*κ*B relative concentration; CRC: caspase-3 relative concentration; AC: apoptosis cells of unit islet area.

NC: normal control group; HF: high-fat diet group; HF+S: high-fat diet plus STZ intraperitoneal injection group; HF+T+S: high-fat diet plus telmisartan intervention and STZ intraperitoneal injection group.

^●^ *P* < 0.05; *P < 0.01 as compared with NC group; ^▲^ *P* < 0.05, ^#^<0.01 as compared with HF group; ^★^<0.05, ^∇^<0.01 as compared with HF+S group.
